# MTHFD2 promotes ovarian cancer growth and metastasis via activation of the STAT3 signaling pathway

**DOI:** 10.1002/2211-5463.13249

**Published:** 2021-09-18

**Authors:** Qiutong Li, Fang Yang, Xiu Shi, Shimin Bian, Fangrong Shen, Yuhong Wu, Chenjie Zhu, Fengqing Fu, Juan Wang, Jinhua Zhou, Youguo Chen

**Affiliations:** ^1^ Department of Obstetrics and Gynecology The First Affiliated Hospital of Soochow University China; ^2^ Clinical Research Center of Obstetrics and Gynecology Jiangsu Key Laboratory of Clinical Immunology Soochow University China; ^3^ Jiangsu Institute of Clinical Immunology The First Affiliated Hospital of Soochow University China; ^4^ Jiangsu Key Laboratory of Clinical Immunology Soochow University China; ^5^ The Second Hospital of Anhui Medical University Hefei China

**Keywords:** cell cycle, EMT progress, methylenetetrahydrofolate dehydrogenase 2, ovarian cancer, STAT3 signaling pathway

## Abstract

Methylenetetrahydrofolate dehydrogenase 2 (MTHFD2) is a bifunctional enzyme located in the mitochondria. *MTHFD2* has been reported to be overexpressed in several malignant tumors and is implicated in cancer development. This study aimed to investigate the effect of MTHFD2 on ovarian cancer progression. The expression of *MTHFD2* was detected by bioinformatic analysis, immunohistochemistry, RT‐qPCR (real‐time quantitative PCR analysis), and western blot analysis. The effects of MTHFD2 depletion on cell proliferation, migration, and invasion were determined through *in vitro* experiments. Cell cycle progression and apoptosis were accessed by flow cytometry. The related signaling pathway protein expression was determined by western blot analysis. We found that *MTHFD2* is highly expressed in both ovarian cancer tissues and cell lines. MTHFD2 deletion suppressed cell proliferation and metastasis. Knockdown of MTHFD2 induces cell apoptosis and G2/M arrest, whereas the number of cells in S phase increased with MTHFD2 overexpression. Mechanically, our results indicate that an inhibitory effect of MTHFD2 knockdown may be mediated by the downregulation of cyclin B1/Cdc2 complex and the inhibitory effect on its activity. Additionally, MTHFD2 could regulate cell growth and aggressiveness via activation of STAT3 and the STAT3‐induced epithelial–mesenchymal transition signaling pathway. These findings indicate that *MTHFD2* is overexpressed in ovarian cancer and regulates cell proliferation and metastasis, presenting an attractive therapeutic target.

## Graphical abstract

Methylenetetrahydrofolate dehydrogenase 2 (MTHFD2) is overexpressed in ovarian cancer tissues and cell lines. MTHFD2 depletion inhibits cell growth and metastasis and induces G2/M arrest and apoptosis in ovarian cancer cell lines. MTHFD2 regulates cyclin B1/Cdc2 complex and activates the STAT3 and STAT3‐induced epithelial–mesenchymal transition signaling pathway. Taken together, our results suggest that targeting MTHFD2 maybe served as a novel approach for ovarian cancer treatment.

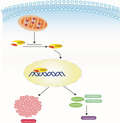

**Abbreviations**
CCK8: cell Counting Kit‐8
EMT: epithelial–mesenchymal transition
MTHFD2: methylenetetrahydrofolate dehydrogenase 2
RT‐qPCR: real‐time quantitative PCR analysis
TMA: tissue microarray

Ovarian cancer is the most common and lethal malignant tumor of the female reproductive system [1]. Due to the asymptomatic nature in the early stage, 75% of patients with ovarian cancer are in the advanced stage at the time of diagnosis [1]. This results in high mortality with only 47% of patients surviving 5 years after diagnosis [2]. Despite advances in the surgical techniques and recent improvements in outcomes due to systemic chemotherapy and poly‐ADP‐ribose polymerase inhibitor maintenance therapy [3, 4, 5], the prognosis of ovarian cancer remains unsatisfactory. Therefore, it is of great clinical value to elucidate molecular mechanisms of ovarian cancer progression to identify more specific therapeutic targets and improve prognosis.

Metabolic reprogramming confers metabolic liabilities, which could be exploited for therapeutic targeting in cancer [6]. One‐carbon metabolism has been proposed as a vulnerability due to its contribution to cancer development [7]. This metabolic pathway generates three main output processes that are important for cancer progression and tumor cell survival: purine/pyrimidine synthesis, DNA and histone methylation, and the maintenance of redox status [8]. Deregulation of one‐carbon metabolism is a possible driver of oncogenesis and cancer progression [9]. Growing evidence has shown that methylenetetrahydrofolate dehydrogenase 2 (MTHFD2), which is a bifunctional enzyme located in the mitochondria and is involved in one‐carbon metabolism, could be a promising anticancer target related to folate metabolism [10]. Recently studies have shown that MTHFD2 was generally overexpressed in many kinds of malignant tumors including acute myeloid leukemia [11], lung carcinoma [12], hepatocellular carcinoma [13], renal cell carcinoma [14], colorectal cancer [15], and breast cancer [16]. Drugs targeting *MTHFD2* have been considered as therapeutic candidates [17, 18]. However, the role of MTHFD2 in ovarian cancer has not been determined.

In the current study, *MTHFD2* was significantly upregulated in ovarian serous carcinoma tissues and ovarian cancer cell lines. MTHFD2 functioned as a critical protumorigenic factor involved in ovarian cancer progression via STAT3 signaling pathway. Our research provides new insight for the treatment of ovarian cancer.

## Materials and methods

### Ovarian cancer cell culture

The human ovarian cancer cell lines SKOV3, OVCAR3, OVCAR5, OVCAR8, A2780, COV413B, and HEY were purchased from ATCC and cultured in RPMI 1640 (HyClone, Logan, UT, USA) supplemented with 10% fetal bovine serum (Biosera, Nuaille, France) and 1% penicillin/streptomycin in a humidified atmosphere with 5% CO_2_ and 95% air at 37 °C.

### Immunohistochemistry staining assay and tissue microarray

The 100 cases of tissue specimens were obtained from the microarray (ovarian cancer tissues No. BC11115d) purchased from Avilabio Biotechnology Co (Xi'an, China). The assay was performed as the previous study [19]. The experiments were undertaken with the understanding and written consent of each subject. The study methodologies were approved by the ethics committee and conformed to the standards set by the Declaration of Helsinki.

### Bioinformatics analysis

The expression of MTHFD2 was analyzed using microarray gene datasets from the Oncomine, TCGA, and GTEx databases. The differential expression of *MTHFD2* mRNA between ovarian serous adenocarcinoma and normal ovarian surface epithelium was determined.

### Transient transfection

SKOV3 and OVCAR8 cells were both transfected with siMTHD2#1 and siMTHFD2#2 purchased from GenePharma (Shanghai, China). The sequences were as follows:

siMTHFD2#1: sense: 5′‐GCAGUUGUGGGAAUCAA CATT‐3′
antisense: 5′‐UGUUGAUUCCCACAACUGCTT‐3′
siMTHFD2#2: sense: 5′‐GCGAGAAUCCUGCAAGU CATT‐3′
antisense: 5′‐UGACUUGCAGGAUUCUCGCTT‐3′
Normal control (nontarget siRNA): sense:5′‐UUCUCC GAACGUGUCACGUTT‐3′
antisense:5′‐ACGUGACACGUUCGGAGAATT‐3′

The overexpressed cells were built by transfected with pcDNA3.1‐MTHFD2(sm)‐Flag‐Homo plasmid purchased from GenePharma. The vector was transfected as normal control. Transfection was performed using Lipofectamine 3000 (Invitrogen, Carlsbad, CA, USA) according to the protocol.

### RNA isolation and real‐time quantitative PCR analysis

Total RNA was extracted with TRIzol reagent (Invitrogen). β‐Actin was used as an endogenous control. Revert Aid First Strand cDNA Synthesis Kits (Thermo Fisher Scientific, Carlsbad, CA, USA) were used for cDNA synthesis. The following PCR primer sequences were used: GATCCTGGTTGGCGAGAATCC (MTHFD2 forward), TCTGGAAGAGGCAACTG (MTHFD2 reverse), AAAGACCTGTACGCCAACAC (β‐actin forward), and GTCATACTCCTGCTTGCTGAT (β‐actin reverse).

RT‐qPCR was performed using Power SYBR Green PCR Master Mix (Takara, Otsu, Shiga, Japan) according to the instructions. The reactions were performed in a CFX96 (Bio‐Rad Company, Shanghai, China), and the reaction was incubated at 95 °C for 10 min, followed by 40 cycles at 95 °C for 15 s, 60 °C for 30 s, and 72 °C for 30 s. Data analyses were performed using the 2^−ΔΔCt^ method.

### Cell Counting Kit‐8 assay

The cells were seeded onto 96‐well plates at 2500 cells/well in 100 μL and transfected, respectively, with siMTHFD2#1, siMTHFD2#2, and normal control siRNA followed by incubation at 37 °C with 5% CO_2_. Then, 10 μL of Cell Counting Kit‐8 (CCK8) solution (CCK8; Dojindo Laboratories, Kumamoto, Japan) was added to each well, followed by incubation for 1 h and determination of the absorbance at 450 nm by enzyme labeling at 24, 48, 72, and 96 h after transfection. The A2780 cells were seeded at 3000 cells/well and, respectively, transfected with Vector and MTHFD2 plasmid for overexpression followed by incubation. The viability of two groups was detected just as the procedure mentioned above.

### EdU assay

Cells (2.5 × 10^4^/well) were seeded onto 24‐well plates and transfected the next day. To detect and mark newly synthesized DNA, 48 h after transfection, the ovarian cancer cells were incubated with 10 μm EdU (Beyotime Institute of Biotechnology, Shanghai, China) for 1 h. Then, the cells were fixed with formaldehyde for 15 min and treated with 0.3% Triton X‐100 for 15 min at room temperature. After three washes with PBS, the cells were treated with 200 μL of 1× Click Additive Solution for 30 min. The DNA in each well was stained with Hoechst33342 (blue) for 10 min and observed under a TCS SP8 confocal microscope (Leica‐Microsystems, Wetzlar, Germany).

### Colony formation assay

The transfected ovarian cancer cells (1000 cells/well) were seeded onto 6‐well plates and incubated in medium for 10–14 days, fixed in 4% paraformaldehyde, and stained with 0.5% crystal violet (Beyotime Institute of Biotechnology). Colonies containing more than 50 cells were counted using a microscope.

### Cell cycle and apoptosis assay

Cells were seeded onto 6‐well plates and transfected with siRNA or plasmid the next day, harvested 72 h after transfections, washed with ice‐cold PBS, fixed in 70% ethanol, and kept at −4 °C overnight. Then, cells were stained with PI/RNase Staining Buffer Solution (BD Biosciences PharMingen, San Diego, CA, USA, No. 550825). The fluorescence intensity of cells was analyzed by flow cytometry FC500/FC500‐MPL (Beckman Coulter, Brea, CA, USA) (Ex = 493 nm and Em = 636 nm). An Annexin V FITC Apoptosis Detection Kit (BD Biosciences PharMingen, No. 556547) was used to distinguish live cells from early apoptotic cells (Annexin V stained) and late apoptotic cells (stained with Annexin V and PI). The transfected cells were harvested and resuspended in 100 µL binding buffer. Then, 5 µL FITC and 5 µL PI were added to each sample and incubated for 15 min in the dark. Finally, the fluorescence intensity of cells was analyzed using flow cytometry.

### Migration and invasion assay

Assays were performed using 24‐well Transwell chambers with (invasion) or without (migration) Matrigel. Five hundred microliter of a serum‐free suspension of cells (6.0 × 10^4^ cells·mL^−1^) was added to the interior of each Transwell insert (pore size, 8 μm), and 500 µL of medium containing 15% FBS was added to the lower chamber of the insert. The cells were incubated at 37 °C in a 5% CO_2_ atmosphere for 24 h (migration) or 36 h (invasion). Then, noninvading cells in the interior of the insert were removed gently with a cotton‐tipped swab. Invasive cells on the lower surface of the inserts were stained and then counted under a microscope.

### Western blot analysis

Equal amounts of lysates (25 µg) were separated using 10% SDS/PAGE electrophoresis, and the separated bands were transferred to polyvinylidene difluoride membranes using 100 V at 4 °C. The membranes were blocked with 5% BSA for 1 h and incubated with antibodies targeting MTHFD2 (1 : 1000; Abcam, Cambridge, UK), α‐tubulin, Cdc2, cyclin B1, p‐STAT3, STAT3, p‐Wee1, Wee1, N‐cadherin, Vimentin (1 : 1000; Cell Signaling Technology, Danvers, MA, USA), and β‐actin antibody (1 : 1000; Sigma‐Aldrich, St. Louis, MO, USA) overnight at 4 °C. Finally, secondary antibodies (MultiSciences, Hangzhou, China) were incubated with membranes for 1 h at room temperature. Antigenic proteins were visualized using an enhanced chemiluminescence system.

### Establishment of stable cell lines

The shMTHFD2 and control shRNA virus were synthesized from GeneChem (Shanghai, China), and cells were transfected with virus according to the manufacture's protocol. Then, the stable MTHFD2 knockdown OVCAR8 and SKOV3 cells were screened and established by using puromycin (Sigma, Burlington, MA, USA) for 7–10 days postvirus transfection.

### *In* *vivo* growth assay

The female BALB/c‐Nude mice (6–8 weeks old) were applied for vivo xenograft research purchased by Shanghai SLAC Laboratory Animal Co (Shanghai, China). The experiments have been approved by the Ethics Committee of Soochow University, and the approved institutional animal use regulations were strictly complied. Mice were housed in the specific pathogen‑free barrier facilities with 35–40% relative humidity and 12‐h light/dark cycle and fed with standard food and drinking water. At the study endpoint, the mice were humanely sacrificed via cervical dislocation. To observe the tumor growth *in vivo*, OVCAR8‐shRNA‐Ctrl and OVCAR8‐shMTHFD2 (4 × 10^6^) cells were injected subcutaneously into the BALB/c‐Nude mice. The Vernier caliper was used to measure the length and width of the tumor three times every 6 days, and the tumor volume was calculated by using the formula length/2 × width^2^ with the average length and width. The experiments have been approved by the Ethics Committee of Soochow University. The animal experiment guide was strictly followed the ‘Laboratory animal—Guideline for ethical review of animal welfare’ (GB/T 35,892–2018).

### Statistical analysis

Each experiment was independently repeated at least three times, and data were analyzed with the statistical program spss (version 22.0, IBM Corp., Armonk, NY, USA). The associations between the expression level of MTHFD2 and clinicopathological parameters in ovarian cancer patients were evaluated using Pearson's chi‐square and Fisher's exact tests. The differences between two groups were analyzed using Student's *t*‐test. graphpad prism 7.0 (San Diego, CA, USA) was used for multiple comparison tests. Results are presented as mean ± standard deviation. Differences were considered significant at *P* < 0.05 (*P < 0.05, **P < 0.01).

## Results

### MTHFD2 is highly expressed in ovarian cancer tissues and cell lines

**Fig. 1:**
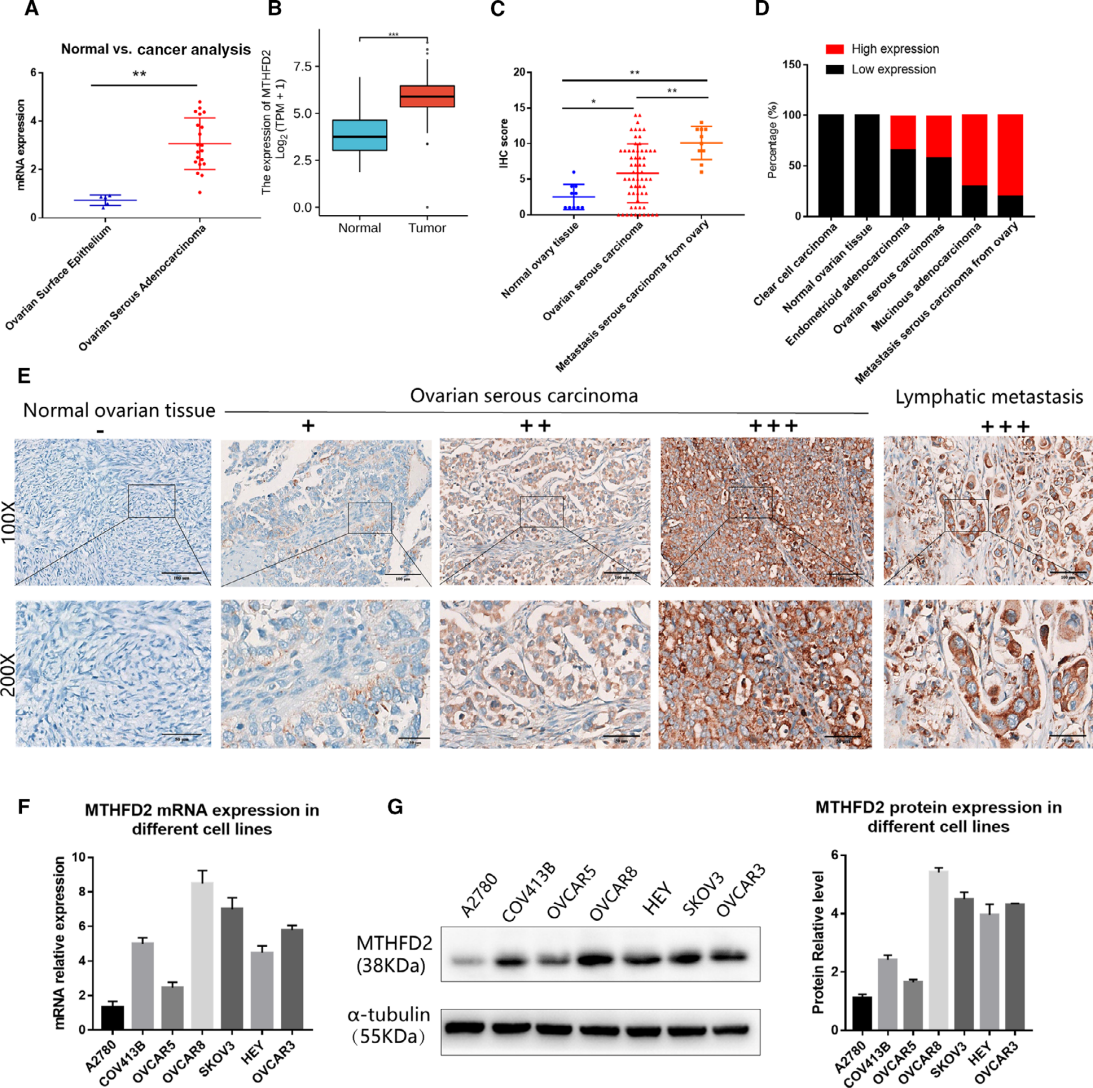
MTHFD2 is highly expressed in ovarian cancer tissues and cell lines. (A) *MTHFD2* mRNA expression in datasets of Lu Ovarian Statistics dataset from Oncomine database. (B) Unpaired tissues of *MTHFD2* expression in TCGA and GTEx databases. (C) The expression of MTHFD2 was examined by IHC. The IHC staining score was higher in ovarian serous carcinoma than in normal ovarian tissues; besides, it was significantly higher in lymph node metastasis carcinomas from ovary. (D) The percentages of tissues with high or low‐expression of MTHFD2 in normal ovarian tissues, ovarian serous carcinoma, mucinous adenocarcinoma, endometrioid adenocarcinoma, clear cell carcinoma, and lymph node metastasis carcinomas. (E) Representative images of MTHFD2 IHC staining in normal ovarian tissue, ovarian serous carcinomas, and lymph node metastasis carcinoma. (−) no staining; (+) weak staining; (++) moderate staining; (+++) strong staining. (F) Expression of *MTHFD2* mRNA in ovarian cancer cell lines as detected by qPCR. (G) Expression of *MTHFD2* protein in ovarian cancer cell lines as detected by western blot. Data were analyzed using Student's *t*‐test. Results are shown as mean ± standard deviation of three independent experiments [Scale bar = 100 μm (100×), Scale bar = 50 μm (50×), *P < 0.05, **P < 0.01, ***P < 0.001].

Bioinformatic analysis of the Oncomine microarray gene expression Lu Ovarian dataset, the unpaired tissues from TCGA and GTEx databases showed that the expression level of MTHFD2 mRNA in ovarian serous adenocarcinoma was significantly higher than normal ovarian surface epithelium (Fig. 1A,B). Besides, the expression of MTHFD2 in ovarian cancer was detected by immunohistochemical analysis using commercially available tissue microarray (TMA). As shown in Fig. 1C, the IHC scores of lymph node metastasis were especially high, and the scores of primary tumors and metastasis were both higher than normal ovarian tissues. And MTHFD2 was highly expressed (++ to +++) in 8/10 (80%) cases of metastasis and in 26/62 (41.94%) cases of ovarian serous carcinoma (Fig. 1D). However, the expression of MTHFD2 was not correlated with age, FIGO staging, and TNM stage (Table S1). The representative immunohistochemistry images were shown in Fig. 1E. All the results above suggest that MTHFD2 may contribute to ovarian cancer development. Therefore, we conducted qPCR and western blot analysis, which indicated that most ovarian cancer cell lines exhibited high MTHFD2 expression (Fig. 1F,G).

### MTHFD2 depletion suppresses the proliferation of ovarian cancer cells *in vitro* and *in vivo*

**Fig. 2:**
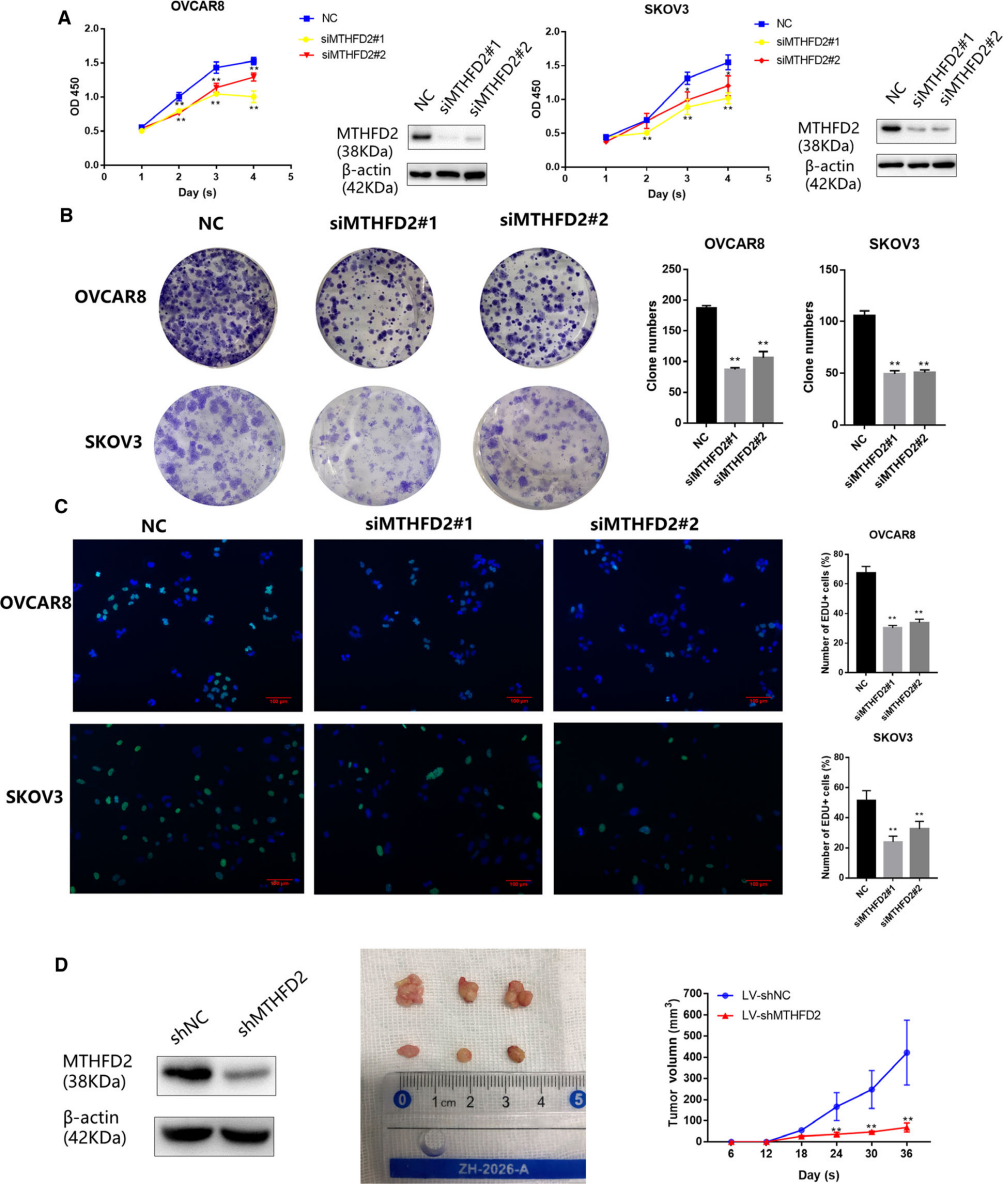
MTHFD2 depletion suppresses the proliferation of ovarian cancer cells *in vitro* and *in vivo*. (A) CCK8 cell viability results in SKOV3 and OVCAR8 cells over 0–96 h after transfection with siMTHFD2#1, siMTHFD2#2, or control siRNA. (B) Representative images of crystal violet staining of colonies of SKOV3 and OVCAR8 cells transfected with the indicated siRNAs after 10–14 days of growth. (C) EdU‐positive cells indicating newly synthesized DNA in ovarian cancer cells transfected with indicated siRNAs. Multiple comparison tests were applied to analyze the data. (D) The curve of tumor volume was measured and analyzed in the LV‐shMTHFD2 group and LV‐shNC group. Data were analyzed using Student's *t*‐test to compare the difference between the two groups. The experiments were repeated three times independently, and the results are shown as mean ± standard deviation (**P < 0.01, green: EdU, blue: Hoechst, Scale bar = 100 μm).

Dysregulated proliferation is a hallmark of tumorigenicity in cancer cells, and thus, we investigated whether MTHFD2 regulates tumor growth. SKOV3 and OVCAR8 cells were transfected with siRNA targeting MTHFD2 (siMTHFD2#1 and siMTHFD2#2). MTHFD2 depletion inhibited cell viability over 0–96 h after transfection compared with the normal control group (Fig. 2A). Colony formation activity was also inhibited (Fig. 2B). Moreover, the EdU assay was used to detect newly synthesized DNA as a marker of proliferating cells. The number of positive staining cells reduced after transfection with MTHFD2 siRNAs (Fig. 2C), indicating that decreased viability was partially due to the impairment of proliferation. We then examined whether MTHFD2‐depleted cells could be less tumorigenic in nude mouse xenografts and observed the suppression of *in vivo* tumor development by MTHFD2 deficiency (Fig. 2D).

### Overexpression of MTHFD2 increases the S phase and promotes proliferation of A2780 cells

To further investigate the effect of MTHFD2 on the proliferation of ovarian cancer cells, CCK8 assay and cell cycle assay were performed after the transfection of MTHFD2 overexpression plasmid and the vector as control group. As shown in Fig. S1A, the viability of cells increased after the overexpression of MTHFD2 compared with the control group. Additionally, after overexpression of MTHFD2, the percentage of S phase was significantly higher (Fig. S1B), indicating that MTHFD2 could promote the proliferation and cell cycle progression of cells.

### MTHFD2 depletion suppresses the migration and invasion of ovarian cancer cells

**Fig. 3:**
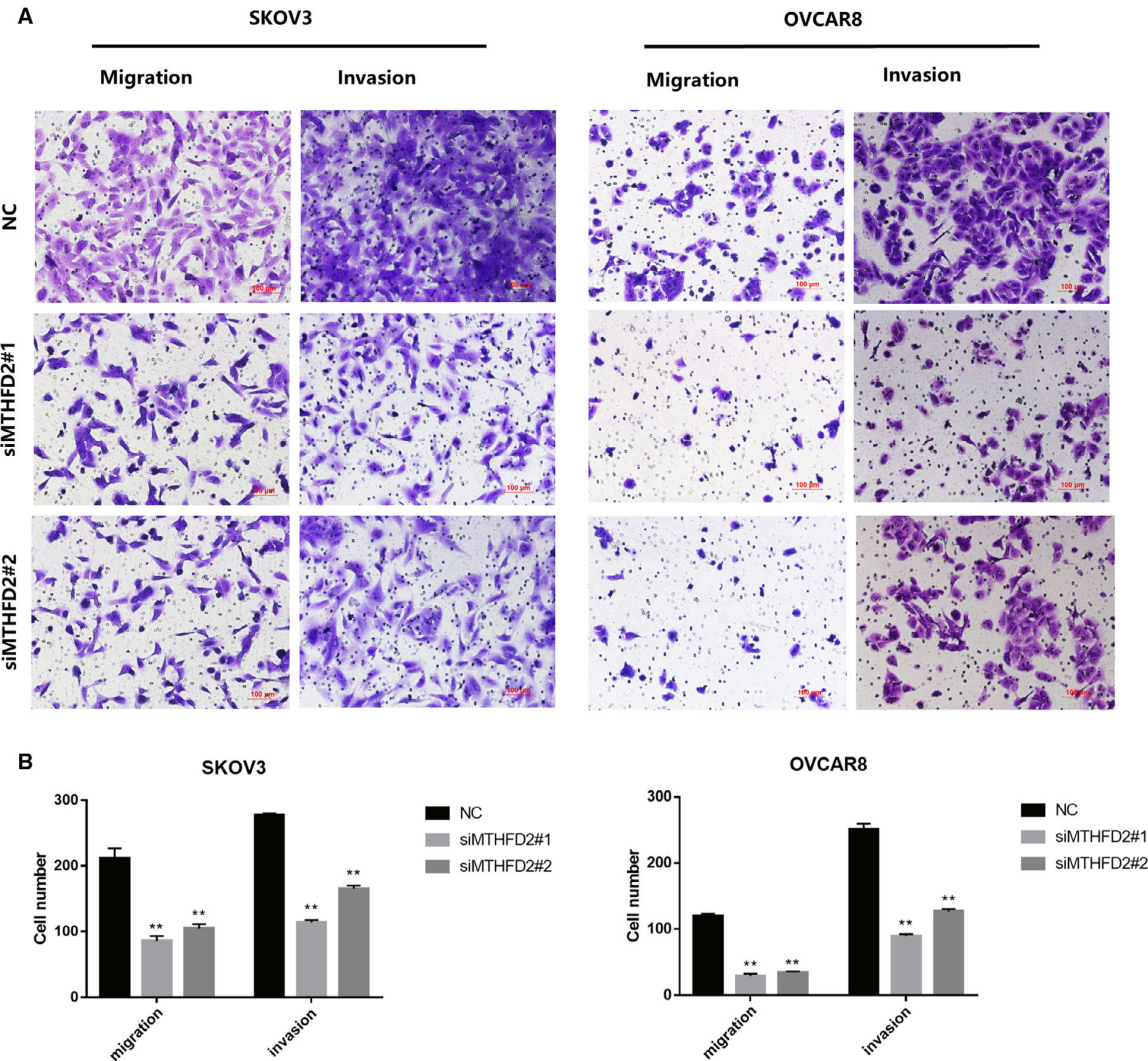
MTHFD2 depletion suppresses the migration and invasion of ovarian cancer cells. (A) Transwell assays to detect effects of MTHFD2 on invasion and migration of SKOV3 and OVCAR8 cells. Images and quantification of migrated and invading ovarian cancer cells transfected with the indicated siRNAs. (B) The results of three independent Transwell assays are analyzed by multiple *t*‐tests and shown as mean ± standard deviation (**P < 0.01, Scale bar = 100 μm).

To evaluate the function of MTHFD2 in ovarian cancer metastasis, Transwell assay was used to detect the tumor aggressiveness. The migration and invasion abilities of siMTHFD2‐transfected SKOV3 and OVCAR8 cells were both significantly suppressed compared with those transfected with control siRNA (Fig. 3A,B).

### Knockdown of MTHFD2 induces G2/M cell cycle arrest and cell apoptosis

**Fig. 4:**
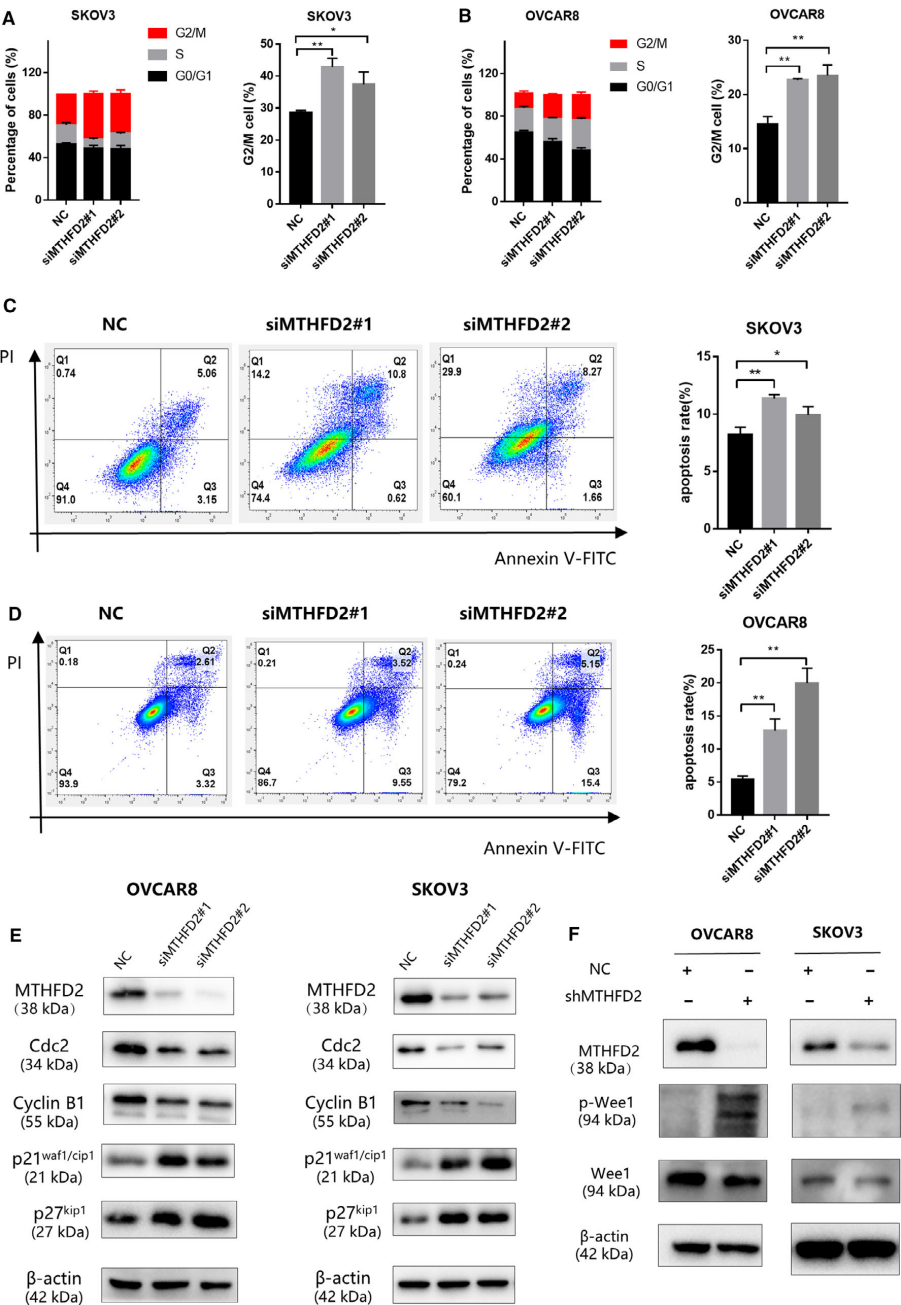
Knockdown of MTHFD2 induces G2/M arrest and cell apoptosis. (A, B) PI staining of SKOV3 and OVCAR8 cells 72 h after transfection with the indicated siRNAs. Flow cytometry analysis shows the cell cycle distribution and G2/M fraction of cells. (C, D) The apoptosis rates of SKOV3 and OVCAR8 cells 72 h after transfection with the indicated siRNAs as evaluated by Annexin V FITC/PI staining. Data of three independent experiments are shown as mean ± standard deviation. (E) The expression of G2/M checkpoint biomarkers, cyclin B1, Cdc2, p21^waf1/cip1^, p27^kip1^, proteins were detected by western blot 72 h after transfection into SKOV3 and OVCAR8 cells. The expressions of cyclin B1 and Cdc2 in the MTHFD2 knockdown groups significantly decreased compared with the normal control group. (F) The expression of p‐Wee1 and Wee1 proteins of shMTHFD2 group and shNC group was detected. The expression of p‐Wee1 in the MTHFD2 silencing group is significantly higher than the normal control group. Multiple comparison tests were applied to compare the difference between the groups (*P < 0.05, **P < 0.01).

Next, to further investigate the mechanism of the inhibitory effect on ovarian cancer cell growth, we postulated that MTHFD2 regulates ovarian cancer cell cycle progression. To verify this hypothesis, we harvested transfected cells and investigate the proportions of cells in various stages of the cell cycle by propidium iodide (PI) staining (Fig. 4A). Compared to the normal control group, an increased percentage of cells in G2/M phase was observed in MTHFD2‐depleted groups. The results demonstrated that MTHFD2 depletion induced G2/M cell arrest (Fig. 4B). Additionally, an increase in apoptosis can also lead to an inhibitory effect on tumor growth. Therefore, Annexin V FITC and PI staining were used to evaluate the apoptosis rate. The percentage of apoptotic cells in MTHFD2‐depleted groups were both higher than the normal control group (Fig. 4C,D).

Mitosis is triggered by progressive activation of the cyclin B1/Cdc2 complex at the end of G2 phase [20]. To investigate whether the cell cycle change upon MTHFD2 depletion was associated with G2/M checkpoint, we detected the expression of characteristic cell cycle promoting complex cyclin B1/Cdc2. The protein level of Cdc2 and cyclin B1 were both decreased with MTHFD2 depletion. Moreover, inhibitory regulators such as p‐wee1, p21^waf1/cip1^, and p27^kip1^ were increased in the MTHFD2 silencing ovarian cancer cells (Fig. 4E,F).

### MTHFD2 promotes ovarian cancer cell progression via STAT3 pathway

**Fig. 5:**
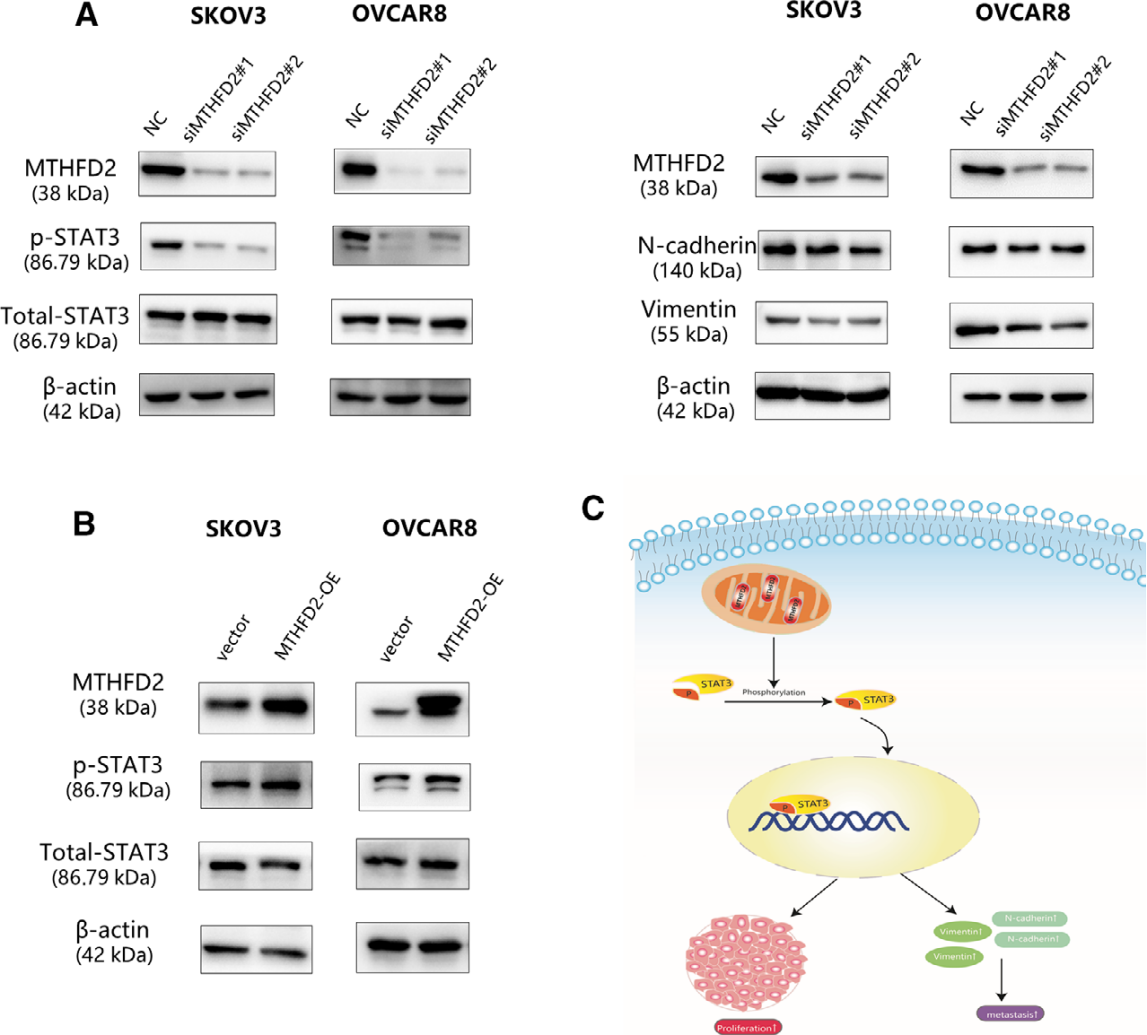
MTHFD2 regulates ovarian cancer cell progression via STAT3 pathway. (A) Protein expression levels of p‐STAT3, STAT3, N‐cadherin, and Vimentin as tested by western blot in the indicated cells with knockdown of MTHFD2 using the indicated siRNAs. (B) Protein expression levels of p‐STAT3 and STAT3 as tested by western blot in SKOV3 cells and OVCAR8 cells with overexpression of MTHFD2 using the plasmid The experiments were repeated three times independently. (C) Schematic diagram of the MTHFD2/STAT3 regulatory pathways in cells.

To understand the underlying molecular mechanisms of MTHFD2‐enhanced proliferation and aggressiveness, we firstly screened the potential correlated proteins with MTHFD2 from the online UCSC database (Fig. S2), among them, the STAT3 signaling pathway which is related to cell growth, metastasis, and apoptosis attracted our attention [18]. Western blot analysis was conducted to test the levels of p‐STAT3, STAT3, and epithelial–mesenchymal transition (EMT)‐related proteins. STAT3 phosphorylation was inactivated by MTHDF2 knockdown in SKOV3 and OVCAR8 cells. Besides, the expression of N‐cadherin and Vimentin, which are important regulatory biomarkers of tumor aggressiveness, significantly reduced upon MTFD2 depletion [21] (Fig. 5A). To verify the correlation of MTHFD2 and STAT3 signaling pathway, the upregulated expression of p‐STAT3 was detected in SKOV3 and OVCAR8 cells with overexpression of MTHFD2 (Fig. 5B). These results indicated that MTHFD2 could promote ovarian cancer cell progression through STAT3 pathway (Fig. 5C).

## Discussion

Methylenetetrahydrofolate dehydrogenase 2 is a NAD+‐dependent enzyme with dehydrogenase and cyclohydrolase activity [22]. Recent studies have demonstrated that overexpression of MTHFD2 was related to poor prognosis in colorectal cancer and lung adenocarcinoma [10, 23]. The expression of MTHFD2 was related to ovarian cancer proliferation. Our results demonstrated that the expression of MTHFD2 was detected in most ovarian cancer cell lines at mRNA and protein levels. According to our bioinformatic analysis and the IHC results of TMA, MTHFD2 is overexpressed in ovarian serous carcinoma and lymphatic metastasis which indicates that MTHFD2 may play an important role in the progression of ovarian cancer.

A previous study showed that MTHFD2 is broadly required for cancer cell proliferation and viability [23]. Stimulation by growth factors can lead to the proliferation of cancer cells, and in parallel with the upregulation of the MTHFD2, which further drives cancer cell proliferation independently of its dehydrogenase activity [24]. Based on gene expression assays, MTHFD2 is associated with both high proliferation rates and cMYC overexpression [25]. The study on NSCLC has shown that MTHFD2 was related to cell growth and tumorigenicity both *in vitro* and *in vivo* by suppressing cycle‐related genes [11, 12]. Another study in colorectal cancer cell lines showed that knockdown of MTHFD2 suppressed cell proliferation and promoted apoptosis [26]. Interestingly, we found similar functions of MTHFD2 in our study. Knockdown of MTHFD2 in ovarian cancer cells reduced the proliferation, induced the G2/M cell cycle arrest and cell apoptosis. Contrarily, overexpression of MTHFD2 promoted cell proliferation and increased the M phases, indicating the cell division ability was enhanced. These results are consistent with our hypothesis that MTHFD2 may be a protumorigenic factor in ovarian cancer.

Dysfunction of the cell cycle plays an essential role in the development of ovarian cancer [27]. The critical G2/M transition of the cell cycle is controlled by the activation of mitosis‐promoting factor, which is composed by cyclin B1/Cdc2 complex [21]. The activation of the complex leads to phosphorylation of numerous substrates essential for mitosis [28]. Decreased accumulation of the complex leads to blocked mitosis and induces G2/M arrest. A prolonged G2 stage can promote apoptosis rather than cell division [29]. Cell proliferation can be repressed by Cdc2 inhibition, which induces G2/M arrest and increased apoptosis in ovarian cancer cell lines [30]. Depletion of cyclin B1 also contributed to the similar effects in various kinds of tumor cells, including HeLa, MCF‐7, and PC‐3 [31]. Our results consistently demonstrated decreased mitotic markers Cdc2 and cyclin B1 upon MTHFD2 depletion. The activation of the cyclin B1/Cdc2 complex is a required biochemical step for mitosis that can be inhibited by p21^waf1/cip1^ and p27^kip1^, two traditional CDK inhibitors. These inhibitors impede growth partially due to the negative regulation of Cdc2 activity [32]. The inhibitor p21^waf1/cip1^ directly interacts with and inhibits cyclin B1/Cdc2 in mitosis [33]. P27^kip1^ also can bind to and inhibit Cdc2 kinase when cells are arrested in the G2/M phase [34, 35]. Additionally, Wee1 protein could schedule the cell division through its inhibitory phosphorylation of conserved tyrosine 15 residues on Cdc2 [36]. The phosphorylation of Wee1 inactivates the Cyclin B1/Cdc2 complex, resulting in G2 arrest [37]. Our results demonstrate the upregulated protein level of p21^waf1/cip1^, p27^kip1^, and p‐Wee1 upon MTHFD2 depletion. Based on these findings, we speculate that MTHFD2 depletion may induce G2/M arrest via downregulation of the cyclin B1/Cdc2 complex formation and activity.

The STAT3 signaling pathway is related to ovarian cancer cell proliferation, metastasis, apoptosis, and differentiation [38]. STAT proteins accelerate EMT progress in several cancer types [39, 40, 41]. Studies in epithelial ovarian cancer have shown the inactivation of STAT3 can reduce N‐cadherin and vimentin expression and suppress cell migration behavior [42, 43]. Consistently, we observed reduced expression of phosphorylated STAT3, N‐cadherin, and Vimentin in MHTFD2 knockdown cells, indicating that the STAT3 signaling‐induced EMT was inhibited. Additionally, the increased p‐STAT3 was found in MTHFD2 overexpressed SKOV3 and OVCAR8 cells. Thus, these results suggested that MTHFD2 depletion suppressed ovarian cancer cell growth and aggressiveness maybe partly due to the inactivation of STAT3 signaling pathway.

Our findings demonstrated that MTHFD2 is upregulated in ovarian cancer tissues and cell lines. MTHFD2 depletion inhibits cell growth and metastasis and induces G2/M arrest and apoptosis in ovarian cancer cell lines. We also found that MTHFD2 could regulate cyclin B1/Cdc2 complex and STAT3 signaling pathway. Taken together, our results indicate an inhibitory effect of MTHFD2 knockdown on tumor development and suggest that targeting MTHFD2 maybe served as a novel approach for effective treatment of ovarian cancer.

## Conflict of interest

The authors declare no conflict of interest.

## Author contributions

QL and FY carried out the experiments, analyzed the data, and drafted the original manuscript. XS and SB assisted with data collection and analysis. YW and CZ participated in experiments. FS and FF conceived of the study and participated in its design and coordination. JW, JZ, and YC supervised the project design and reviewed the manuscript. All authors read and approved the final manuscript.

## Supporting information

**Fig. S1.** Overexpression of MTHFD2 increased the S phase and promotes proliferation of A2780 cells. A. CCK8 cell viability results in A2780 cells over 0–120 h after transfection with vector and plasmid for overexpression of MTHFD2. B. PI staining of A2780 cells 72 h after transfection with the plasmid for overexpression. Flow cytometry analysis shows the cell cycle distribution and the increased S phase. Student's t‐test was applied to compare the difference between the two groups. Data of three independent experiments were shown as mean ± SD (**P < 0.01).
**Fig. S2.** The potential correlated proteins with MTHFD2 screened by the UCSC online database.
**Table S1.** Correlation of MTHFD2 expression by immunohistochemistry with clinicopathological parameters in ovarian cancer patients. The expression level of MTHFD2 and clinicopathological parameters in ovarian cancer patients were evaluated using Pearson's chi‐square and Fisher's exact tests.

## Data Availability

The data that support the findings of this study are available from the corresponding author on reasonable request.
