# Supplementary material for: MTHFD2 promotes ovarian cancer growth and metastasis via activation of the STAT3 signaling pathway
Source: FEBS Open Bio. 2021 Sep 18;11(10):2845–57. doi: 10.1002/2211-5463.13249 (PMC8487042; doi:10.1002/2211-5463.13249)

**Supplementary**

**Supplementary Table 1**.

Correlation of MTHFD2 expression by immunohistochemistry with clinicopathological parameters in ovarian cancer patients

| Clinical  parameters | Cases (n) | MTHFD2 expression (%) | | P-value |
| --- | --- | --- | --- | --- |
|  |  | Low | High |  |
| Age (years) | | | | |
| ≥50 | 51 | 28 (54.90) | 23 (45.10) | 0.549 |
| ＜50 | 49 | 30 (61.22) | 19 (38.78) |  |
| FIGO staging | | | | |
| I | 48 | 28 (58.33) | 20 (41.67) | 0.476 |
| II | 14 | 10 (71.43) | 4 (28.57) |  |
| III | 15 | 7 (46.67) | 8 (53.33) |  |
| IV | 3 | 1 (33.33) | 2 (66.67) |  |
| T stage | | | | |
| T1 | 48 | 28 (41.67) | 20 (58.33) | 0.182 |
| T2 | 15 | 11 (73.33) | 4 (26.67) |  |
| T3 | 17 | 7 (41.17) | 10 (58.83) |  |
| N stage | | | | |
| N1 | 12 | 5 (41.67) | 7 (58.33) | 0.229 |
| N0 | 68 | 41(60.29) | 27 (39.71) |  |
| M stage | | | | |
| M1 | 3 | 1 (33.33) | 2 (66.67) | 0.388 |
| M0 | 77 | 45 (58.44) | 32 (41.56) |  |
| Pathology diagnosis | | | | |
| Normal ovarian tissue | 10 | 10 (100.00) | 0 (0.00) | 0.000** |
| Ovarian serous carcinoma | 62 | 36(58.06) | 26 (41.94) |  |
| Clear cell carcinoma | 5 | 5 (100.00) | 0 (0.00) |  |
| Mucinous adenocarcinoma | 10 | 3 (30.00) | 7 (70.00) |  |
| Endometrioid adenocarcinoma | 3 | 2 (66.67) | 1 (33.33) |  |
| Metastasis serous carcinoma from ovary | 10 | 2 (20.00) | 8 (80.00) |  |

MTHFD2: Methylenetetrahydrofolate dehydrogenase 2

FIGO staging: The Ovarian Cancer Staging System of International Federation of Gynecology and Obstetrics in 2014. (*, *P*＜0.05, ** *P*＜0.01)

Supplementary Figure 1. Overexpression of MTHFD2 increased the S phase and promotes proliferation of A2780 cells. A. CCK8 cell viability results in A2780 cells over 0–120 h after transfection with vector and plasmid for overexpression of MTHFD2. B. PI staining of A2780 cells 72 h after transfection with the plasmid for overexpression. Flow cytometry analysis shows the cell cycle distribution and the increased S phase. Data of three independent experiments are shown as mean ± SD. (**P＜0.05, **P＜0.01*)


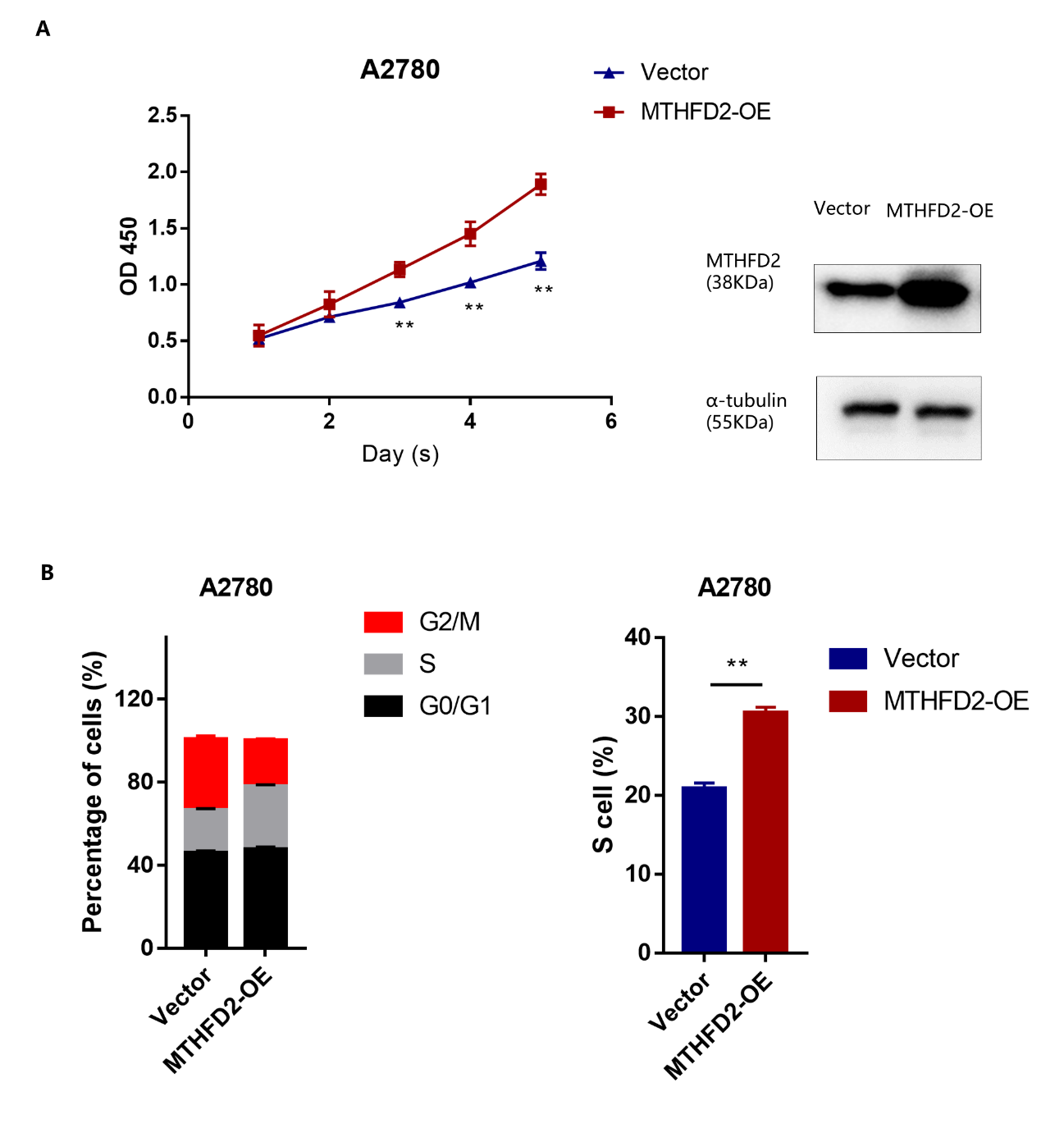


Supplementary Figure 2. The potential correlated proteins with MTHFD2 screened by the UCSC online database.


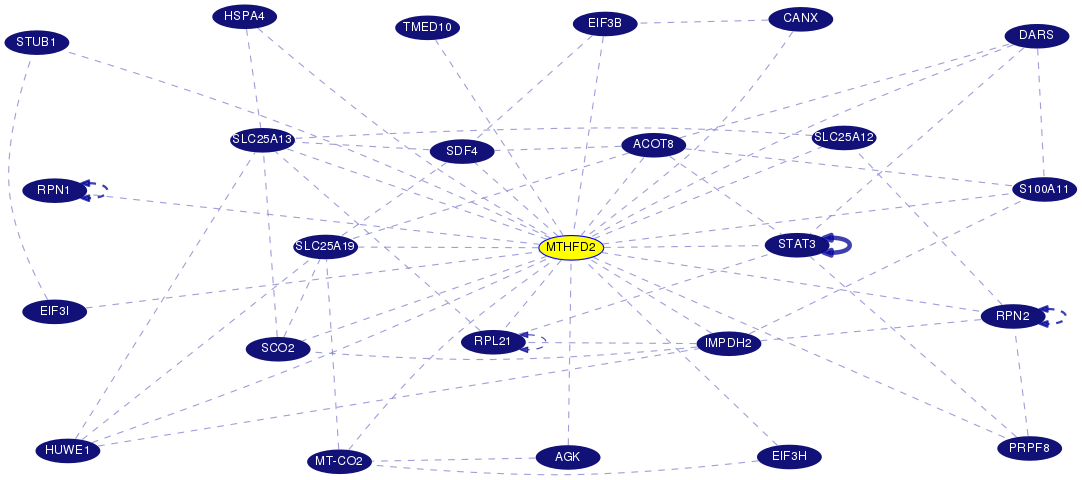

Supplement: Supplementary file 1 — Fig. S1. Overexpression of MTHFD2 increased the S phase and promotes proliferation of A2780 cells. A. CCK8 cell viability results in A2780 cells over 0–120 h after transfection with vector and plasmid for overexpression of MTHFD2. B. PI staining of A2780 cells 72 h after transfection with the plasmid for overexpression. Flow cytometry analysis shows the cell cycle distribution and the increased S phase. Student's t‐test was applied to compare the difference between the two groups. Data of three independent experiments were shown as mean ± SD (**P < 0.01). Fig. S2. The potential correlated proteins with MTHFD2 screened by the UCSC online database. Table S1. Correlation of MTHFD2 expression by immunohistochemistry with clinicopathological parameters in ovarian cancer patients. The expression level of MTHFD2 and clinicopathological parameters in ovarian cancer patients were evaluated using Pearson's chi‐square and Fisher's exact tests. [file FEB4-11-2845-s001.docx]
